# Mpox infection presenting with morbilliform rash: A case series

**DOI:** 10.1016/j.jdcr.2023.03.001

**Published:** 2023-03-15

**Authors:** Erin Bartholomew, Cory Kosche, Kieron S. Leslie

**Affiliations:** Department of Dermatology, University of California, San Francisco, San Francisco, Californi

**Keywords:** cutaneous mpox infection, morbilliform exanthem, mpox virus, viral coinfections

## Introduction

Mpox, formerly referred to as monkeypox, is a rare zoonotic disease caused by infection with the mpox virus, a member of the orthopox family along with smallpox and cowpox. Mpox was first discovered and isolated in 1958 and was first diagnosed in a human in 1970 in the Democratic Republic of Congo.^1^

Prior to the ongoing 2022 mpox outbreak, the virus had only been reported in association with Central and Western African countries where the disease was endemic. Previously, mpox cases in humans outside of Africa were almost entirely linked to travel to endemic countries or through imported animals.^2^^,^^3^ At the time of submission, the ongoing epidemic boasted nearly 76,000 cases over 100 countries.^4^ Currently, nearly 75,000 confirmed cases are in locations that have not historically reported mpox infections.^4^ Symptoms of mpox infection often include characteristic umbilicated papules with pustular appearance; however, atypical presentations have been reported.^5^

In the current outbreak, symptoms of mpox infection have been variable and far more diverse than in previous outbreaks. The presentation of mpox includes a flu-like prodrome with fever and malaise followed by lymphadenopathy and the development of a classic human mpox rash.^4^ The rash often begins as a nonspecific papule that enlarges into an umbilicated pustule and spreads centrifugally. Within 14 to 21 days of developing the rash, the lesions ulcerate, subsequently crust over, and eventually re-epithelialize.^4^

However, atypical rashes are now seen in patients with confirmed mpox. Here, we present 4 patients infected with the mpox virus, as confirmed by polymerase chain reaction (PCR) of lesional swabs, in whom an acute morbilliform rash developed in addition to the classic mpox lesions. It is imperative that providers are aware of the uncommon features of mpox infection so that they can maintain a high index of suspicion.

## Case series

**Fig 1:**
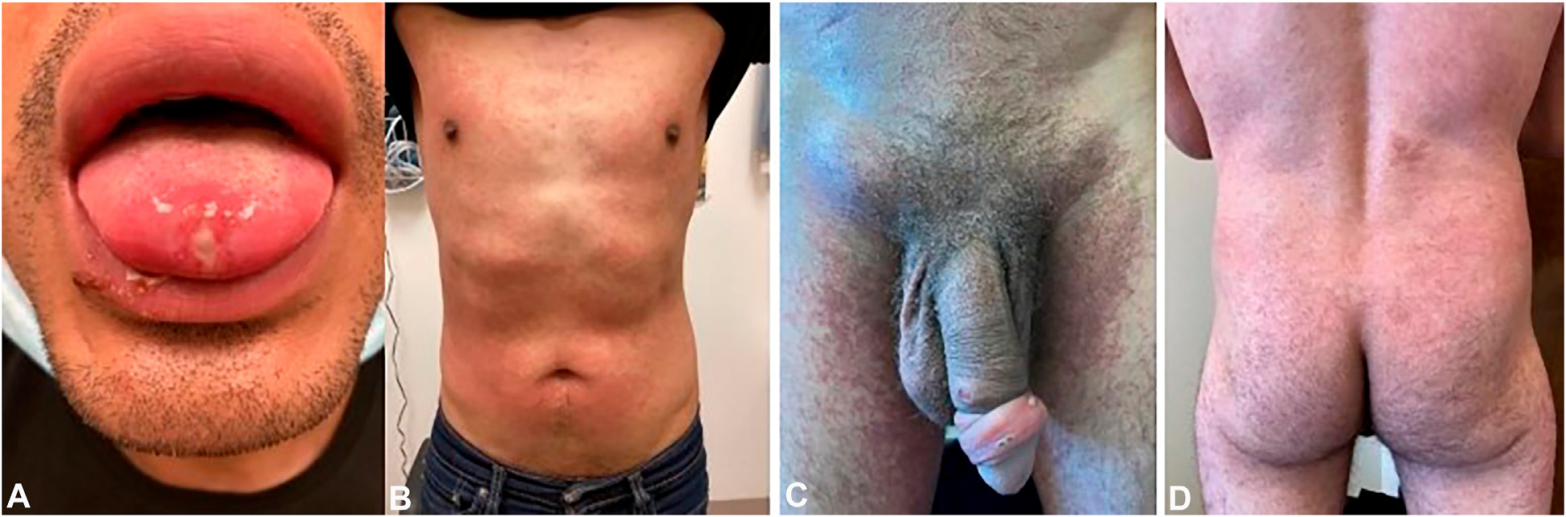
**A,** Patient 1: tender, ulcerated, mpox polymerase chain reaction–positive lesion on the anterior aspect of the tongue. **B,** Patient 1: morbilliform rash. **C,** Patient 4: crusted papules on the penis with associated edema and phimosis. **D,** Patient 4: morbilliform rash on the back and buttocks.

Patient 1 is in his 30s and is a man who has sex with men (MSM). He has no significant medical history. He presented to a sexual health clinic in San Francisco with 1 week of fevers, malaise, and headache followed by the development of a tender ulceration on the anterior aspect of the tongue (Fig 1, *A*); 3 small umbilicated papules on the face, neck, and arm; and a diffuse, pruritic morbilliform rash that favored the trunk and did not involve the palmar or plantar skin (Fig 1, *B*). He denied any recent new medication. His oral lesion was swabbed for mpox, and he was tested for chlamydia, gonorrhea, syphilis, and HIV. He was treated empirically with tecovirimat. Mpox PCR returned a positive result. His rapid plasma reagin (RPR) was reactive at 1:4 dilution. Previously, patient 1 had an RPR titer of 1:1 with no reported history of syphilis. Urine and rectal swabs were positive for *Neisseria gonorrhoeae*. Rapid HIV was negative, and HIV-1 RNA PCR was undetectable. He was treated with intramuscular (IM) penicillin and IM ceftriaxone, notably after the rashes had developed. Both rashes resolved over the next 2 weeks.

Patient 2 is an MSM in his 40s with a medical history of well-controlled HIV, treated Hepatitis C infection, and methamphetamine use who presented to a community clinic in San Francisco with 3 days of fever and myalgias and 1 day of painful ulcerated papules on the scalp and in the perianal region with oral ulcers on the lingual frenulum. He also had a pruritic morbilliform rash on his trunk and reported severe rectal pain. He denied any recent new medications. His HIV was well controlled with an undetectable viral load 1 month prior and a CD4 count of 718 cells/mm^3^. Patient 2 had no prior RPR titers performed or available to our knowledge. He was tested for mpox, gonorrhea, and chlamydia. He was treated empirically with tecovirimat and was administered empiric ceftriaxone and doxycycline. Mpox PCR returned a positive result. Chlamydia and gonorrhea were negative in 3 sites. Lesions had crusted over at his 12-day follow-up appointment, and the morbilliform rash had resolved.

Patient 3 is an MSM in his 30s with a history of well-controlled HIV who presented to an outside clinic with 1 day of fevers and scattered erythematous papules. He was treated empirically with IM penicillin given the concern that a recent partner tested positive for syphilis. The next day, the papules progressed into large umbilicated papules involving the trunk, extremities, face, and perirectal area, and a diffuse, nonpruritic morbilliform rash began to develop. The rash favored the trunk and proximal extremities, coalescing into a large plaque along the back, and did not involve the palmar or plantar skin. He experienced severe rectal pain that led him to present to the emergency department. He denied any other recent new medications. He was tested for mpox and syphilis and was treated empirically with tecovirimat. Mpox PCR returned a positive result. RPR was positive at a 1:32 titer, which is a functionally negative result and unchanged from an RPR collected 1 year prior. He reported good adherence to his antiretrovirals, and on admission, his CD4 count was 1115 cells/mm^3^with an HIV viral load of 256. His morbilliform rash self-resolved in 1 week, around the same time that his classic mpox lesions crusted over.

Patient 4 is an MSM in his 30s with a history of well-controlled HIV who presented to an outpatient HIV dermatology clinic with 2 weeks of painful umbilicated papules on the penis (Fig 1, *C*) and perianal skin and 2 days of a diffuse morbilliform rash (Fig 1, *D*). His penile lesions were associated with penile edema and phimosis. Three days prior to presentation, a transient subjective fever developed, followed by a morbilliform rash coalescing into plaques over the trunk and proximal extremities. It was mildly pruritic. There were no scale and no palmar or plantar lesions, but tender inguinal lymphadenopathy was present. He denied any new recent medications. He was adherent to his antiretrovirals, and his HIV viral load was undetectable 1 day prior to presentation. His last CD4 count 1 year prior was 422 cells/mm^3^. He was tested for mpox, syphilis, chlamydia, and gonorrhea. He was treated with empiric tecovirimat. Mpox testing returned a positive result. RPR was positive at a 1:512 dilution. Patient 4 had an RPR titer of 1:4 6 months prior; 2 years prior, he had an RPR titer of 1:16 and was treated with subsequent improvement. Test results for pharyngeal and rectal chlamydia were positive, and the test result for rectal gonorrhea was positive. The urine test result was negative for both chlamydia and gonorrhea. He was treated with IM penicillin, ceftriaxone, and doxycycline. At the 1-week follow-up, his lesions were improving and the morbilliform rash was resolving.

## Discussion

Classic morbilliform rashes often occur in the setting of a new drug exposure or as a viral exanthem. Morbilliform drug–induced exanthem is the most common drug hypersensitivity reaction, characterized by a disseminated, symmetric eruption of erythematous macules and/or papules that occur approximately 1 to 2 weeks after initiating treatment with the causative drug or, in previously sensitized individuals, as early as 6 to 12 hours and up to 3 days after initiating treatment with the causative drug. The most common eliciting drugs include penicillins, cephalosporins, and agents with sulfhydryl groups. These drugs act as haptens, bind to endogenous proteins, and then become antigens.^6^

Morbilliform viral exanthem in the setting of mpox infection is not a well-known occurrence, although morbilliform eruptions have also been reported as prodromal eruptions in smallpox, a related orthopoxvirus.^7^ To our knowledge, the first case report published in Spain by de Nicolas-Ruanes et al^8^ in June 2022 described a case of mpox virus with maculopapular exanthem and proctitis. In addition, Català et al^9^ published a prospective cross-sectional study of 185 patients with cutaneous mpox findings. In this study, 6% of their population experienced morbilliform rashes during the disease process.^9^ Tarín-Vicente et al^10^ described an observational prospective cohort study that found mpox infection to be the culprit of genital, perianal, and oral lesions. Notably, Tarín-Vicente et al^10^ reported that 4% of the cohort experienced maculopapular exanthem, of whom 3% were ruled as having a morbilliform drug eruption. Only 1 patient in the cohort was reported to have a viral exanthem.

In our case series, we report 4 patients with morbilliform rashes occurring at or around the time of umbilicated papule formation, not associated with the prodromal phase (Table I). Limitations of our observation include the concomitant diagnoses made at the time of mpox diagnosis. However, we do not believe that the morbilliform rashes present are consistent with those typically seen in secondary syphilis because secondary syphilis involves a papulosquamous eruption on the trunk with a predilection for acral areas.^11^^,^^12^

**Table I:** Patient characteristics and diagnostic information

Patient ID	Age decade	Sex	Orientation	Race or ethnicity	HI V status	HIV viral load (copies/mL)	CD4 count (cells/mm^3^)	New medications	Prodrome	Prodrome to rash on set	Mpox treatment	Concomitant testing performed	Concomitant diagnoses	Concomitant disease treatment
Patient 1	30	Male	MSM	Hispanic	Negative	Not applicable	Not applicable	None	No prodrome	1 wk	Tecovirimat	Mpox, RPR, urine GC/CT, HIV	Syphilis (RPR reactive at 1:4), gonorrhea	IM ceftriaxone, IM penicillin
Patient 2	40	Male	MSM	White	Positive	Undetectable	718	None	Fever with myalgias	3 d	Tecovirimat	Mpox, urine GC/CT	None	Prophylactic IM ceftriaxone, PO doxycycline
Patient 3	30	Male	MSM	Hispanic	Positive	256	1115	None	Fever	1 d	Tecovirimat	Mpox and RPR	Syphilis (RPR reactive at 1:32)	Prophylactic IM penicillin
Patient 4	40	Male	MSM	Hispanic/Latino	Positive	Undetectable	422	None	Transient subjective fever	3 d	Tecovirimat	Mpox, RPR, urine, pharyngeal, and rectal GC/CT	Pharyngeal and rectal chlamydia, rectal gonorrhea	Prophylactic IM penicillin, IM ceftriaxone, doxycycline by mouth

*GC/CT*, Gonorrhea/chlamydia; *IM*, intramuscular; *MSM*, men who have sex with men; *PO*, by mouth; *RPR*, rapid plasma reagin.

The rapid development of the rash at the time of mpox symptom onset is also suggestive of a unifying viral-mediated process.

We have described 4 cases of morbilliform rash that are likely related to acute mpox infection. In our patients, the morbilliform rash appeared to occur concomitantly or soon after development of the classic mpox lesions and not as part of the prodrome. All patients had a morbilliform rash presentation with truncal predominance, extremity involvement, and no facial involvement. Patient 4 was the exception whose presentation included striking groin and proximal thigh involvement.

Awareness of this presentation is extremely important so that providers maintain a high index of suspicion for mpox when patients present with a morbilliform exanthem during the current epidemic. The high rate of coinfection with other sexually transmitted infections underscores the importance of broad testing when patients present with symptoms of mpox.

## Conflicts of interest

None disclosed.
